# Longitudinal relations between interpartner aggression and internalizing symptoms among couples: The moderating role of sleep

**DOI:** 10.1111/jsr.14013

**Published:** 2023-08-12

**Authors:** Ryan J. Kelly, Brian T. Gillis, Mona El-Sheikh

**Affiliations:** 1 University of New Mexico, Albuquerque, New Mexico, USA; 2 Auburn University, Auburn, Alabama, USA

**Keywords:** anxiety, depression, intimate partner violence, mental health, romantic relationships, victimization

## Abstract

Recipients of interpartner aggression often experience internalizing symptoms. However, individual differences exist, and elucidation of factors that attenuate or exacerbate risk are needed to explicate relations and better inform interventions aimed at reducing mental health sequelae of interpartner aggression. Sleep problems compromise coping abilities and are known to exacerbate risk for mental health problems in the context of family risk. We examined whether sleep problems moderated the extent to which the recipients of interpartner aggression experience internalizing symptoms over time. At the first wave, 194 couples participated (M age [women] = 41.81 years, SD = 5.85; M age [men] = 43.75 years, SD = 6.74; 71% White/European American, 26% Black/African American, 3% other race/ethnicity). Two years later, couples returned for a second wave. Psychological and physical forms of interpartner aggression were measured using self- and partner-reports. Sleep duration (minutes) and sleep quality (efficiency) were derived using actigraphy, and subjective sleep/wake problems were also assessed. Individuals self-reported on their own internalizing symptoms. After controlling for autoregressive effects, sleep moderated the extent to which the recipients of interpartner aggression experienced internalizing symptoms longitudinally. Lower sleep efficiency and more subjective sleep/wake problems among women exacerbated the extent to which interpartner aggression forecasted their internalizing symptoms. Lower sleep efficiency among men magnified relations between interpartner aggression and their internalizing symptoms. Findings help understand the multiplicative influence that family risk and sleep problems have on mental health over time.

## 1 | INTRODUCTION

Interpartner aggression (IPA) is prevalent among middle-aged couples in long-term relationships (Vickerman & Margolin, 2008). IPA may be psychological (verbal threats, insults, name calling) and physical (assault on partner’s body), and the recipients of such aggression are at risk for internalizing problems including symptoms of depression and anxiety (for review, see Lagdon et al., 2014). However, individual differences exist, and examinations of factors that attenuate or exacerbate risk are needed to better inform interventions aimed at reducing mental health sequelae of IPA (Chuang et al., 2012). Bioregulatory processes including sleep may modify this association and warrant investigation.

Sleep problems compromise daily functioning and inhibit coping abilities, and are known to exacerbate risk for mental health problems in the context of family risk (Troxel, 2010). Along this line, we assessed whether sleep moderates the extent to which the recipients of IPA experience internalizing symptoms over time among a community sample of middle-aged couples. We measured multiple sleep parameters (Sadeh, 2015), including actigraphy-based sleep duration (minutes) and quality (efficiency), and self-reported sleep/wake problems. Sleep problems refer to shorter or poorer-quality sleep derived by actigraphy, and self-reports of more sleep/wake problems (e.g. difficulty falling asleep).

Studies have demonstrated sleep problems as predictors and sequelae of psychological and physical IPA (Gordon & Chen, 2014; Rauer & El-Sheikh, 2012). Further, transactional dynamics and reciprocal associations have been demonstrated between sleep and internalizing symptoms (Alvaro et al., 2013). In addition to these patterns of relations, there are other less-studied dynamics between these variables. Troxel’s conceptual framework pertaining to the interface between relationships, sleep and health, suggested that sleep may moderate the magnitude of relations between IPA and mental health (Troxel, 2010). This type of interaction can be understood within multiplicative risk and diathesis stress models. Multiplicative risk (sometimes called cumulative risk) occurs when one risk factor (e.g. IPA) interacts with another (e.g. sleep problems) to elevate the likelihood for negative outcomes (e.g. internalizing symptoms; Evans et al., 2013). Similarly, diathesis stress occurs when risk from an existing condition is exacerbated by a second condition (Sameroff, 1983). Sleep problems result in a loss of top-down prefrontal regulation of the amygdala, as well as cortical processing in the salience network including the insula and cingulate cortex (Simon et al., 2020; Walker & van der Helm, 2009). These neurological effects increase vulnerability for emotional reactivity, poor impulse control and lower tolerance to frustration, which may inhibit the ability to cope with negative dimensions of relationship functioning including IPA (Killgore et al., 2008; Troxel, 2010).

A small but growing literature has considered interactions between sleep and family conflict and stress; however, most attention has focused on youth (Chiang et al., 2017; El-Sheikh et al., 2014). In those studies, sleep exacerbated the magnitude of relations between family conflict and adjustment problems. For example, parental IPA more robustly predicted adolescents’ aggression for those with shorter sleep duration (Lemola et al., 2012). Although studies with adults are limited, existing evidence is compelling. For example, of mothers 3–6-months postpartum, the strength of associations between negative social interactions, including interpartner conflict and internalizing symptoms was greater for those who self-reported lower sleep quality (Lillis et al., 2018). Collectively, these studies illustrate that sleep may impact the extent to which family and interpartner conflict confer risk, and are consistent with our assessment of sleep as a moderating variable of relations between IPA and internalizing symptoms.

## 2 | CURRENT STUDY

We examined sleep as a moderator of relations between being the recipient of IPA and internalizing symptoms. Our investigation focused on middle-aged couples in long-term relationships, which is important. This population commonly reports IPA and there is a need to elucidate factors that exacerbate or attenuate the extent to which IPA confers risk during this later period of development (Vickerman & Margolin, 2008). Moreover, middle-aged adults are particularly susceptible to poor sleep due to a range of factors including job, career and family responsibilities (Lee et al., 2017; Sheehan et al., 2019). Most studies focused on the recipients of IPA have considered women, yet IPA against men is common (Lagdon et al., 2014). We assessed IPA against men and women. As part of a rigorous investigation, we used a two-wave longitudinal design to explicate whether sleep modulates associations between IPA and change in internalizing symptoms over time.

Given past research showing that sleep problems modulate the influence of family risk, we hypothesized that sleep problems would exacerbate the magnitude of relations between IPA and internalizing symptoms. Given the dearth of prior research, we had no expectations of whether results would be more evident for women or men or whether certain sleep parameters would be more influential.

## 3 | METHODS

### 3.1 | Participants

Data are from the Family Stress and Youth Development Study at Auburn University. Data were utilized from the 4th and 6th waves, collected in 2012–2013 and 2014–2015, respectively, and referred to hereafter as T1 and T2. At the initial wave of the larger study (2005), families were recruited through letters distributed at public schools in the southeastern USA. Inclusion criteria at initial recruitment were parental cohabitation for at least 2 years and having a child in 2nd or 3rd grade. At T1 of the current study, 194 mixed-sex couples participated (*n* = 188 women, 162 men; M length of cohabitation = 15.19 years [SD = 7.52 years]; 95% were married; Table 1). Among 29 couples, only the woman participated. Among three couples, only the man participated. The mean age was 41.81 years for women (SD = 5.85) and 43.75 years for men (SD = 6.74). Further, 71% were non-Hispanic White/European American and 26% were non-Hispanic Black/African American; the remaining 3% of participants reported other races or ethnicities, including *n* = 4 mixed race/ethnicity, *n* = 3 American Indian/Native American, *n* = 1 Hispanic, and *n* = 1 who reported their race/ethnicity as “other”. Socioeconomic status (SES) was measured using income-to-needs ratio, which is the quotient of a family’s total income from all sources divided by the federal poverty threshold for their household size (U.S. Department of Commerce, 2012). Most individuals were of middle class (M = 2.60, SD = 1.30; range = 0.14–6.72).

About 2 years later (M = 1.97 years, SD = 1.04 months), 149 women and 128 men participated at T2 (79% retention rate; Table 1). In addition, four women and 11 men who were part of the larger study but did not participate at T1 were part of the analytical sample at T2. We assessed whether retained and attrited individuals differed on study variables; women who discontinued participation were younger (*t*_185_ = −2.38, *p* = 0.02), of lower SES (*t*_180_ = −3.45, *p* = 0.001), had lower sleep efficiency (*t*_139_ = −3.63, *p* = 0.002) and more sleep/wake problems (*t*_159_ = 2.13, *p* = 0.04). Men who discontinued participation were from lower SES backgrounds (*t*_155_ = −2.22, *p* = 0.03), experienced more IPA (*t*_155_ = 2.59, *p* = 0.01) and had more sleep/wake problems (*t*_123_ = 2.15, *p* = 0.04). Path models used to examine research questions were refit while excluding those who discontinued participation at T2; results were identical in nature to those based on the full sample.

### 3.2 | Procedures

The study was approved by the university’s Institutional Review Board. Participants provided consent. At T1, individuals completed questionnaires online and at about the same time wore actigraphs on their non-dominant wrists while sleeping for seven consecutive nights. At T2, participants completed questionnaires.

### 3.3 | Measures

#### 3.3.1 | Interpartner aggression (T1)

The IPA was measured using the Revised Conflict Tactics Scale (Straus et al., 1996), which is well established and has demonstrated good psychometric properties. Participants reported on the frequency of their own aggression in the past year using the Psychological Aggression subscale (eight items, including: “I shouted or yelled at my partner”; *α* = 0.77–0.79 for women and men) and Physical Aggression subscale (12 items, including: “I pushed or shoved my partner”; *α* = 0.81–0.91). Participants also reported on their partner’s perpetration of aggression using reworded equivalents to reduce potential bias from under- or overestimation in reporting. Response choices ranged from 0 (This has never happened) to 6 (More than 20 times in the past year). Items were summed to create a total score for each scale. Psychological and physical aggression have an additive influence on mental health (Lagdon et al., 2014), and it is recommended that they are examined together (Rabin et al., 2009). In addition, sensitivity of measures is increased when perpetrators’ and recipients’ reports are assessed (Rabin et al., 2009). Rates of physical aggression were low (20% of women and 21% of men experienced physical IPA, average incidents per year was < 1) and similar to those observed in other community samples (Pollard et al., 2023).

Scales were composited in three steps. First, women’s reports of their perpetration of Physical Aggression and Psychological Aggression were summed (*r* = 0.46); the same was done for men (*r* = 0.60). Then, women’s reports of their partner’s perpetration of Physical Aggression and Psychological Aggression against them were summed (*r* = 0.48), and the same was done for men’s reports (*r* = 0.58). Finally, IPA against women was calculated by averaging women’s reports of their partner’s aggression and men’s reports of their own aggression (*r* = 0.63), and IPA against men was calculated as the mean of men’s reports of their partner’s aggression and women’s reports of their own aggression (*r* = 0.60). Compositing of recipients’ and perpetrators’ reports is recommended to account for any potential non-concordance between such reports, especially at higher levels of aggression (Marshall et al., 2011).

#### 3.3.2 | Sleep actigraphy (T1)

Sleep was measured using Octagonal Basic Motionlogger actigraphs (Ambulatory Monitoring, Ardsley, NY, USA), and scored in Action W2 software (Ambulatory Monitoring, 2011). To corroborate actigraphy-derived sleep times, participants completed daily logs (Acebo & Carskadon, 2001). Two established sleep parameters were included in analyses: sleep minutes (total number of minutes spent asleep between sleep onset and wake time) and sleep efficiency (% of minutes scored as sleep between onset and wake time). Consistent with the scoring manual, sleep onset time was determined as the first of at least three consecutive minutes scored as sleep, and wake time was the last of at least five consecutive minutes before waking. Data were captured in 1-min epochs using zero-crossing mode and were analysed with the Cole–Kripke scoring algorithm (Cole et al., 1992). The average across available nights was used. Consistent with best practices (Meltzer et al., 2012), data were treated as missing for those with < 5 nights (*n* = 51 women [27%] and 43 men [25%]). Reasons for missing data included omission of nights on which medication for acute illnesses was reported, forgetting to wear the actigraph, device malfunction, and when self-reported sleep times differed by > 30 min from actigraphy-derived data (this was rare). Rates of missingness are similar to those in other studies (Saini et al., 2021).

#### 3.3.3 | Subjective sleep/wake problems (T1)

Participants reported on sleep/wake problems using the 19-item Pittsburgh Sleep Quality Index (PSQI; Buysse et al., 1989). The PSQI has demonstrated good psychometric properties with community samples (Buysse et al., 1989). The PSQI assesses several dimensions of sleep, including duration, quality, latency and disturbances. The overall global sleep composite was used (possible range = 0–21). About 68% of women and 54% of men scored ≥ 5, indicative of poor sleep in at least two domains (Buysse et al., 1989; *α* = 0.72).

#### 3.3.4 | Internalizing symptoms (T1 and T2)

Participants reported on their internalizing symptoms over the previous 2 weeks using the Symptom Checklist-90-Revised (Derogatis, 1983), a survey of symptoms of psychopathology with strong psychometric properties (Olsen et al., 2004). The Anxiety (10 items; e.g. “Feeling so restless you couldn’t sit still”; *α* = 0.86–0.94) and Depression (13 items; e.g. “Feeling no interest in things”; *α* = 0.91–0.93) subscales were used. Response choices ranged from 0 (not at all) to 4 (extremely). Anxiety and depression have overlapping aetiologies, especially within community samples, and their unitary response to treatment supports their consideration as a single cluster of symptoms (Persons et al., 2003). The anxiety and depression subscales were highly correlated (*r* = 0.78–0.86 at T1 and 0.82–0.85 at T2), and were summed to derive the internalizing symptoms variable at each wave.

#### 3.3.5 | Covariates

We controlled for variables known to relate to sleep and adjustment, including age, SES and body mass index (BMI) at T1. BMI was calculated from self-reported weight and height, using the formula of weight (in kilograms) divided by height squared (in metres). We also controlled for race/ethnicity; for both women and men, two dummy variables were created: 0 = White, 1 = Black; 0 = White, 1 = Other (e.g. Hispanic, American Indian/Native American).

### 3.4 | Plan of analysis

We first fit path models to assess the direct relations between IPA and sleep at T1 and internalizing symptoms at T2. Separate models were fit for women and men. The three sleep parameters were included simultaneously, which allowed for examining one sleep parameter while controlling for the others. Overall, two models were fit to assess direct associations, one for women and one for men. Internalizing symptoms were controlled at T1; this approach helps reduce bias in parameter estimates, allows for conclusions about predicted change, and provides information about directionality of associations (Selig & Little, 2012).

**FIGURE 1:**
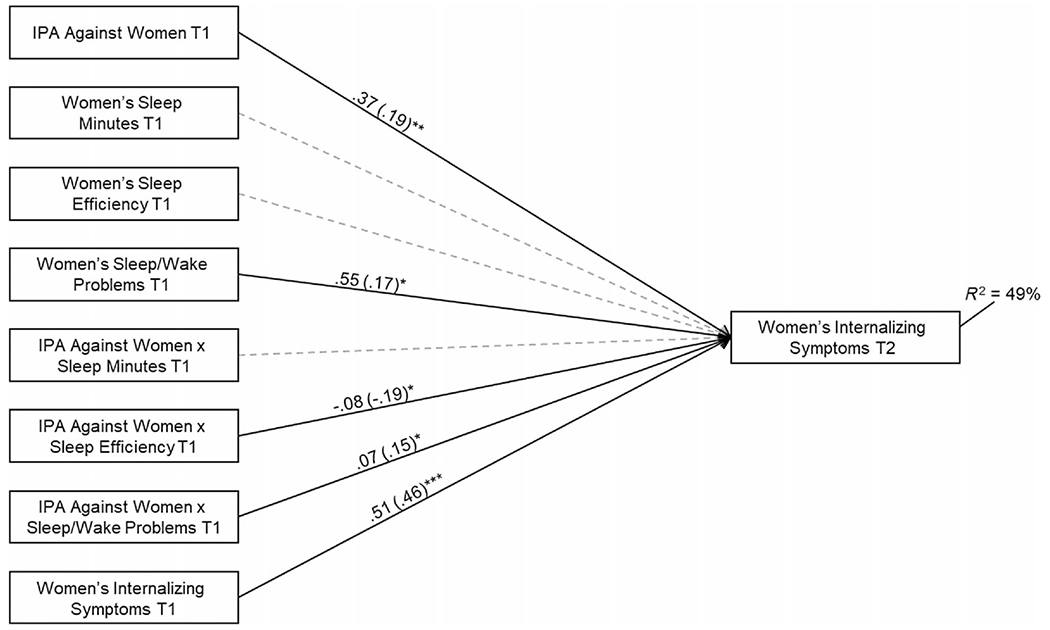
Examination of women’s sleep at T1 as a moderator of relations between interpartner aggression (IPA) against women at T1 and women’s internalizing symptoms at T2. Women’s race, age, body mass index (BMI) and family socioeconomic status (SES) at T1 were included as control variables (the covariates and their associated paths are not depicted for clarity). Statistically significant lines are solid and non-significant lines are dotted. Model fit: *χ*^2^(48) = 51.61 ns; CFI = 0.97; RMSEA = 0.04 ns, 95% confidence interval (CI) [0.00–0.07]. Exogenous variables that were significantly related were allowed to covary. Unstandardized and standardized coefficients (in parentheses) are included (*p < 0.05. **p < 0.01. ***p < 0.001).

**FIGURE 2:**
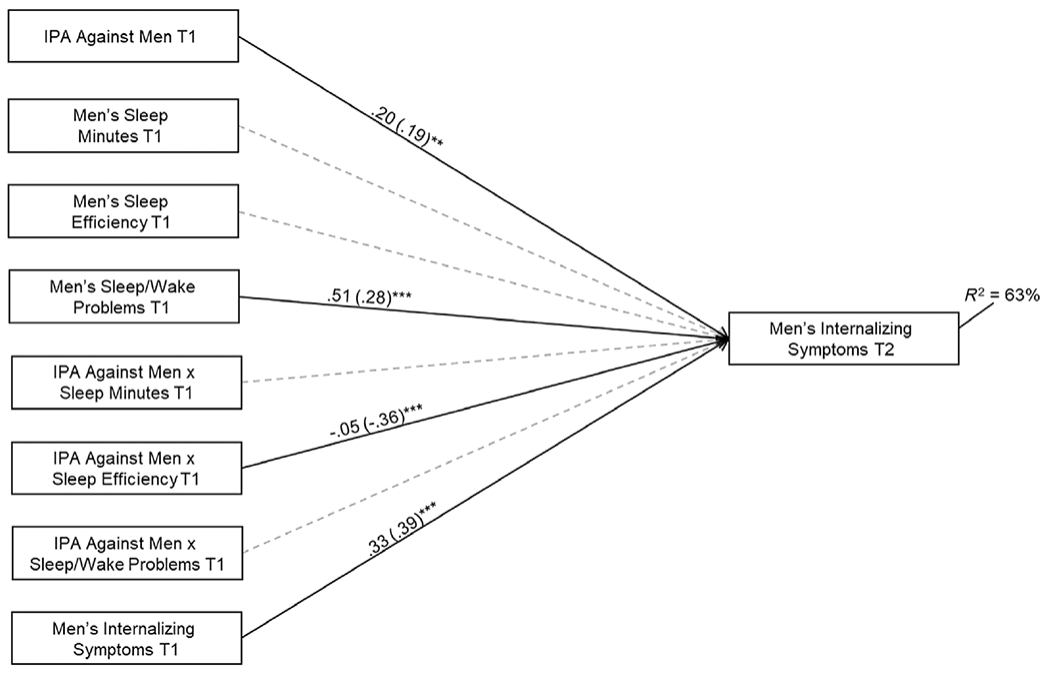
Examination of men’s sleep at T1 as a moderator of relations between interpartner aggression (IPA) against men at T1 and men’s internalizing symptoms at T2. Men’s race, age, body mass index (BMI) and family socioeconomic status (SES) at T1 were included as control variables (the covariates and their associated paths are not depicted for clarity). Model fit: *χ*^2^(44) = 30.98 ns; CFI = 1.00; RMSEA = 0.00 ns, 95% confidence interval (CI) [0.00–0.04]. Exogenous variables that were significantly related were allowed to covary. Unstandardized and standardized coefficients (in parentheses) are included (**p < 0.01; ***p < 0.001).

Next, three interactions were added to each of the two models (Figures 1 and 2) to examine whether sleep moderated relations between IPA and internalizing symptoms over time. Each interaction involved one of the sleep parameters. In preliminary analyses, we fit separate models for each interaction term, and results were nearly identical compared with the full models. Interactions were plotted using Preacher et al.’s (2006) online utility. Following established recommendations, interactions were plotted at high and low (± 1 SD) levels of IPA and sleep (Cohen et al., 2003).

Given that participants were in relationships, we considered actor–partner interdependence models (APIM) to assess research questions because of their ability to account for the interdependence of data (Kenny, 2018). Although this approach entails in the examination of partner effects and the estimation of paths non-pertinent to research questions, APIMs were fit in exploratory analyses. The findings were identical to those reported in the Results (both direct relations and moderation results), and are included in Supporting Information. Thus, when interdependence was taken into consideration, findings did not change.

Models were fit using Amos 24. Significant relations among exogenous variables were estimated. The covariates were allowed to predict internalizing symptoms at T2. Participants had between 73% and 93% of data across primary variables (IPA, sleep parameters, internalizing symptoms). We conducted independent samples *t*-tests to assess mean differences on primary variables based on missingness; no significant differences emerged. Full-information maximum likelihood was used to handle missing data. To reduce the influence of outliers, high-leverage values surpassing 3 SDs were replaced at the highest observed value below 3 SDs (Cousineau & Chartier, 2010); between 5 and 11 values were recoded across variables. Skew was examined using visual observation and kurtosis and skewness statistics (± 2); no variables were skewed. Multiple model fit indices were reported, including *χ*^2^, CFI, RMSEA; all models fit the data well.

## 4 | RESULTS

### 4.1 | Preliminary analyses

Descriptive statistics and bivariate correlations are presented in Table 2. About 80% of women and 81% of men experienced at least one form of IPA at T1. Based on actigraphy at T1, women’s average sleep-onset time was 23:06 hours (SD = 86 min), and morning wake time was 06:13 hours (SD = 106 min). Men’s average sleep-onset time was 23:33 hours (SD = 166 min), and morning wake time was 06:36 hours (SD = 179 min). On average, women slept for 6 hr and 40 min per night (SD = 59.38 min), and men slept for 6 hr and 5 min per night (SD = 72.91 min); hours refer to actual sleep during the sleep period minus night awakenings. Sleep duration, bedtimes and wake times were analogous to similar populations (Duncan et al., 2016). Average sleep efficiency in our sample was 95.23% for women and 91.69% for men, which reflects high levels of efficiency.

Testing of mean differences indicated that women were younger than men (*t*_190_ = −5.20, *p* < 0.001). Compared with women, men were the recipients of more IPA (*t*_184_ = −3.16, *p* = 0.002), had shorter sleep (*t*_106_ = 4.00, *p* < 0.001) and poorer sleep efficiency (*t*_106_ = 4.68, *p* < 0.001). Women reported more internalizing symptoms than men at T2 (*t*_101_ = 2.67, *p* = 0.009).

### 4.2 | IPA against women and internalizing symptoms over time: The role of women’s sleep

A model was fit to assess the direct relations between IPA against women, women’s sleep and internalizing symptoms at T2, *χ*^2^(28) = 21.22 ns; CFI = 0.99; RMSEA = 0.00 ns, 95% confidence interval (CI) [0.00–0.06] (not depicted in figure for brevity). Of the covariates, only women’s internalizing symptoms at T1 and T2 were related (*B* = 0.50, *β* = 0.44, *p* < 0.001). Higher IPA predicted increases in internalizing symptoms over time (*B* = 0.35, *β* = 0.18, *p* = 0.01). More sleep/wake problems at T1 predicted more internalizing symptoms at T2 (*B* = 0.63, *β* = 0.19, *p* = 0.01).

**FIGURE 3:**
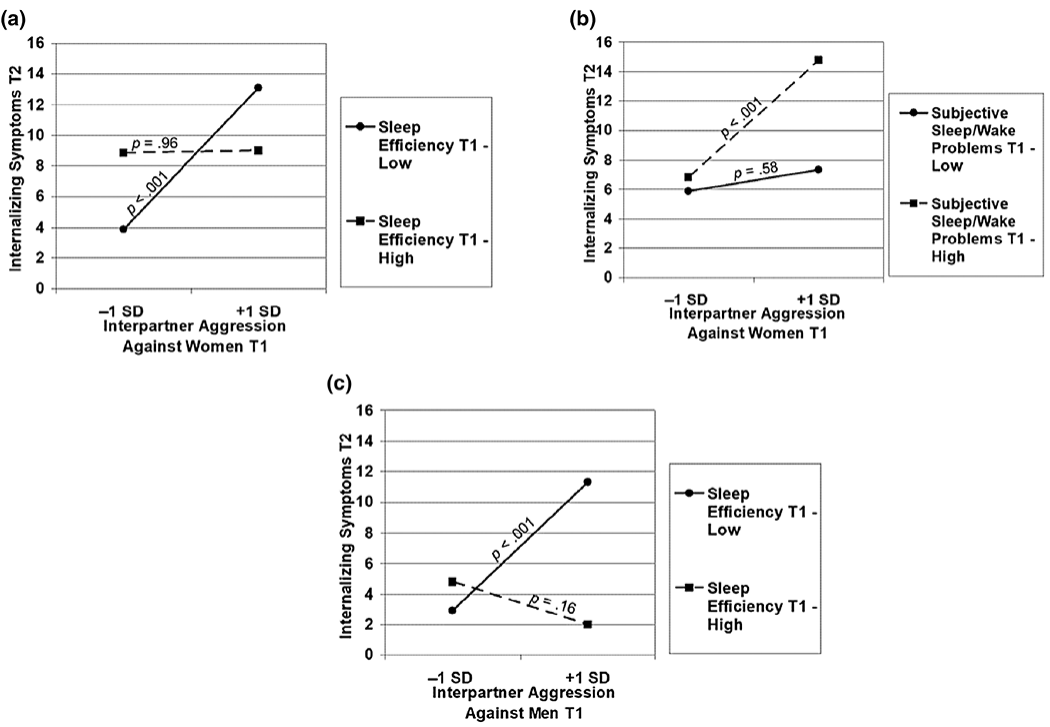
(a) Sleep efficiency at T1 as a moderator of relations between interpartner aggression (IPA) against women at T1 and their internalizing symptoms at T2. (b) Subjective sleep/wake problems at T1 as a moderator of relations between IPA against women at T1 and their internalizing symptoms at T2. (c) Sleep efficiency at T1 as a moderator of relations between IPA against men at T1 and their internalizing symptoms at T2.

Next, interaction terms were added to examine sleep as a moderator, χ^2^(48) = 51.61 *ns*; CFI = 0.97; RMSEA = 0.04 ns, 95% CI [0.00–0.07]. Women’s sleep efficiency at T1 emerged as a moderator (Figure 1). IPA forecasted greater internalizing symptoms for those with lower sleep efficiency (Figure 3a; predicted M = 3.86 and 13.08 at lower and higher levels of IPA, respectively). Based on the regions of significance, this association was significant for those with sleep efficiency < 94.23 (*n* = 42; i.e. those with efficiency < 0.21 SD below the mean). The simple slope was not significant for those with higher sleep efficiency who tended to have relatively average levels of internalizing symptoms regardless of IPA (predicted M = 8.86 and 9.00 at lower and higher levels of IPA, respectively).

The interaction between women’s sleep/wake problems and IPA was significant (Figure 1). IPA was related to internalizing symptoms for women with greater sleep problems (Figure 3b; predicted M = 6.85 and 14.76 at lower and higher levels of IPA, respectively). The association was significant for those with sleep/wake problems > 5.53 (*n* = 90; i.e. those with sleep problems 0.27 SD ± the mean). The simple slope was not significant for women with fewer sleep problems who tended to have fewer internalizing symptoms regardless of IPA (predicted M = 5.88 and 7.31 at lower and higher levels of IPA, respectively).

### 4.3 | IPA against men and internalizing symptoms over time: The role of men’s sleep

A model was fit to assess the direct relations between IPA against men, men’s sleep internalizing symptoms, *χ*^2^(31) = 16.13 ns; CFI = 1.00; RMSEA = 0.00 ns, 95% CI [0.00–0.04] (model not depicted in figure). Internalizing symptoms at T1 and T2 were associated (*B* = 0.44, *β* = 0.51, *p* < 0.001). Age was negatively related to internalizing symptoms (*B* = −0.02, *β* = −0.21, *p* = 0.003). More IPA and sleep/wake problems at T1 were related to more internalizing symptoms at T2 (*B* = 0.19, *β* = 0.18, *p* = 0.01 and *B* = 0.37, *β* = 0.20, *p* = 0.02, respectively).

Interaction terms were added, *χ*^2^(44) = 30.98 ns; CFI = 1.00; RMSEA = 0.00 ns, 95% CI [0.00–0.04] (Figure 2). Demonstrative of moderation, IPA predicted internalizing symptoms for those with lower sleep efficiency (Figure 3c; predicted M = 2.92 and 11.30 at lower and higher levels of IPA, respectively). The association was significant for those with efficiency < 92.44 (*n* = 48; < 0.09 SD above the mean). The simple slope was not significant for those with higher efficiency (predicted M = 4.79 and 1.98 at lower and higher levels of IPA, respectively).

## 5 | DISCUSSION

We examined sleep as a moderator of relations between IPA and internalizing symptoms over 2 years. We recruited a relatively large sample of couples, assessed sleep using actigraphs (sleep duration, efficiency) and self-reports (sleep/wake problems), and controlled for earlier levels of internalizing symptoms. Our investigation included men, which is uncommon in this literature (Hines & Douglas, 2009). Multiple sleep–wake problems exacerbated relations between IPA and increases in internalizing symptoms over time. Findings build on the literature to consider sleep in the context of romantic relationships (Gordon & Chen, 2014; Rauer & El-Sheikh, 2012), and demonstrate the conjoint role of IPA and sleep problems on mental health.

Interpartner aggression was related to increases in internalizing symptoms for women and men. However, sleep modified this association. Supportive of hypotheses, lower sleep efficiency and more sleep/wake problems among women exacerbated the impact of IPA on internalizing symptoms. The influence of IPA was less evident when women’s sleep was more optimal. Similarly, poorer sleep efficiency exacerbated relations between IPA and internalizing symptoms among men. For women and men, the patterns were consistent such that the multiplicative impact of IPA and sleep problems predicted increases in internalizing symptoms.

The findings build on an emerging literature to report interactions between familial risk and sleep. However, most studies have focused on children and adolescents, and inquiries involving adults and relationship discord are scarce (Chiang et al., 2017; El-Sheikh et al., 2014). The consideration of adult couples in long-term relationships is important. Relationship discord occurs often in this population, and there is a need to better elucidate factors that exacerbate the negative influence of IPA on mental health trajectories during this later developmental period (Vickerman & Margolin, 2008). The results are among the first to show that sleep problems confer risk for internalizing symptoms in the context of IPA. Continued examinations of the synergy between sleep and romantic relationship functioning hold promise for elucidating a key dynamic process in health-risk trajectories in midlife.

The observed moderation findings are in line with Troxel’s (2010) proposition that relations between conflict in romantic relationships and mental health can be better understood in the context of sleep. The results are also consistent with multiplicative risk (Evans et al., 2013) and diathesis stress models (Sameroff, 1983), such that interactions between one risk factor (i.e. IPA) and another (i.e. sleep problems) were associated with greater likelihood of a negative outcome (i.e. internalizing symptoms). Sleep problems compromise functioning in the prefrontal cortex and amygdala, as well as cortical processing in the salience network including the insula and cingulate cortex (Simon et al., 2020; Walker & van der Helm, 2009), which in turn may impact self-regulation and impede the ability to cope with relationship discord (Killgore et al., 2008; Troxel, 2010). In contrast, more optimal sleep may prevent deterioration of stress resistance and preserve mental health among those experiencing conflict in their relationships. In addition, findings fit within a broader literature suggesting that both psychological stress and sleep problems lead to allostatic load (McEwen & Karatsoreos, 2015), contributing to dysregulation in stress response systems with negative effects on health and well-being (Benham, 2010). For example, sleep problems lead to dysregulation of the hypothalamic–pituitary–adrenocortical axis, which involves increased cortisol secretion, and may exacerbate the health risks of IPA (Buckley & Schatzberg, 2005; Omisade et al., 2010).

Sleep efficiency and sleep/wake problems, but not sleep duration, interacted with IPA. Other work yielded similar results, such that sleep quality but not duration moderated relations between family risk and youths’ developmental outcomes (Chiang et al., 2017; El-Sheikh et al., 2014). It is not clear why this pattern emerged; however, poor sleep quality in particular can disrupt prefrontal cortex functioning and the ability to cope with social stress (Walker & van der Helm, 2009), and this could have contributed to the findings. Moreover, our findings indicated that relatively high levels of sleep efficiency were needed to mitigate the magnitude of relations between IPA and internalizing symptoms for women (sleep efficiency > 94.23%) and men (sleep efficiency > 92.44%). IPA is a major stressor and robust predictor of internalizing symptoms, and it is possible that particularly high levels of sleep efficiency are required to prevent a deterioration of stress resistance and to preserve mental health.

Low and non-significant correlations were observed between actigraphy and self-reports of sleep. Other studies with community samples have reported similar discrepancies (Saini et al., 2021), and plausible reasons exist. What constitutes “good” sleep varies across individuals (Hirshkowitz et al., 2015), and some participants who obtained poorer actigraphy-derived sleep may still have reported their sleep to be adequate (El-Sheikh et al., 2014). Further, sleep is multifaceted (Sadeh, 2015), and the self-report instrument tapped into some sleep parameters not measured with actigraphy (e.g. difficulty falling asleep, daytime sleepiness). Thus, individuals who reported various sleep-related problems may have still experienced longer and higher-quality actigraphy-derived sleep. Overall, findings illustrate the importance of assessing several parameters using different measures.

In addition to the observed interactions, IPA and sleep were related in other ways. Correlational analyses revealed that women and men who experienced IPA reported more sleep/wake problems, which mirrors other results with community samples (Rauer & El-Sheikh, 2012). It is possible that stress from IPA contributes to sleep problems and vice versa. Overall, dynamics between IPA and sleep are complex, and these variables may be associated in multiple ways.

The study includes implications. In our community sample, most participants experienced at least one form of IPA. Mitigating IPA in communities is challenging; however, prevention programs have reported encouraging results, and continued efforts are warranted (Cummings et al., 2008). Further, results may help inform interventions aimed at reducing mental health sequelae of IPA and highlight the importance of considering sleep. Multiple variables may be targeted to modify sleep, including sleep schedules, physical activity and sleeping conditions (e.g. noise, temperature; Novak & Gillis, 2022).

This study includes limitations. We focused on important sleep parameters, yet others (e.g. sleep stages, chronobiology) may be relevant. We aggregated milder and more severe forms of IPA, and future research should clarify whether results vary by type or severity. Given the community sample and relatively mild IPA and sleep problems in our study, results do not necessarily generalize to clinical samples. Those who discontinued at T2 differed on some variables. Models were refit while excluding those who discontinued participation, and results were similar in nature to those based on the full sample. Nevertheless, the pattern of attrition could still have impacted the results. Further, while sleep tends to be stable over 2 years in middle adulthood (Liu et al., 2016), it cannot be conclusively stated that only T1 sleep moderates associations between IPA at T1 and internalizing symptoms at T2, as sleep at T2 could also play a role. The larger study from which our data were drawn did not measure sleep at T2, and thus this could not be assessed.

The present study used a rigorous design, and provided novel insight into factors that modify relations between IPA and internalizing symptoms while highlighting the pivotal role of sleep. Continued investigations of sleep as a moderator are likely to add new understanding of the nature of relations between dimensions of romantic relationship functioning and mental health.

## Supplementary Material

Supporting Material

## Figures and Tables

**TABLE 1:** Sociodemographic characteristics of sample

	T1		T2	
	Women (*n* = 188)	Men (*n* = 162)	Women (*n* = 149)	Men (*n* = 128)
Age M (SD)	41.81 (5.85)	43.75 (6.74)	44.11 (5.78)	46.33 (6.30)

Race/ethnicity				
Black/AA *n* (%)	49 (27%)	38 (25%)	35 (24%)	32 (25%)
White/EA *n* (%)	131 (72%)	109 (71%)	111 (75%)	93 (74%)
Others *n* (%)	3 (2%)	6 (4%)	2 (1%)	1 (1%)

Income-to-needs ratio M (SD)	2.60 (1.30)		3.52 (1.86)	

Length of cohabitation M (SD)	15.19 (7.52)		20.21 (6.93)	

Married couples *n* (%)	182 (95%)		140 (88%)	

*Note*: Age and length of cohabitation reported in years.

Abbreviation: AA, African American; EA, European American.

**TABLE 2:** Descriptive statistics and correlations for main study variables

	M	SD	Min.	Max.	1	2	3	4	5	6	7	8	9	10	11	12
1. IPA against women at T1	6.12	6.50	0.00	30.50	–											
2. IPA against men at T1	6.84	6.97	0.00	31.00	0.90***	–										
3. Women’s sleep minutes at T1 (in hr)	6.67	0.99	3.89	9.30	−0.08	−0.05	–									
4. Women’s sleep efficiency at T1	95.23	4.67	79.06	99.91	−0.07	−0.08	0.37***	–								
5. Women’s subjective sleep/wake problems at T1	6.58	3.83	0.00	18.00	0.21**	0.27***	−0.12	0.24**	–							
6. Men’s sleep minutes at T1 (in hr)	6.08	1.22	2.86	8.48	0.01	0.01	0.33***	0.06	−0.02	–						
7. Men’s sleep efficiency at T1	91.69	8.06	62.56	99.79	0.04	−0.05	0.25*	0.32***	−0.02	0.56***	–					
8. Men’s subjective sleep/wake problems at T1	5.78	4.01	0.00	17.00	0.30***	0.26**	0.04	0.04	0.21*	−0.08	−0.06	–				
9. Women’s internalizing symptoms at T1	8.65	11.30	0.00	44.00	0.37***	0.39***	0.02	−0.10	0.49***	−0.02	−0.01	0.18*	–			
10. Men’s internalizing symptoms at T1	5.79	8.71	0.00	34.56	0.33***	0.29***	−0.04	−0.01	0.27**	0.05	0.00	0.53***	0.25**	–		
11. Women’s internalizing symptoms at T2	8.27	11.50	0.00	45.00	0.41***	0.40***	−0.13	−0.06	0.48***	−0.14	−0.13	0.16	0.67***	0.26**	–	
12. Men’s internalizing symptoms at T2	4.67	7.34	0.00	34.00	0.37***	0.43***	−0.11	0.07	0.25*	−0.05	−0.04	0.42***	0.34***	0.62***	0.38***	–

*Note*: For ease of interpretation, sleep duration is reported in hours; minutes were used in analyses.

Abbreviation: IPA, interpartner aggression.

* *p* ≤ 0.05.

** *p* ≤ 0.01.

*** *p* ≤ 0.001.

## Data Availability

Data are not available yet for sharing with others. Per National Institutes of Health data sharing guidelines, they will be available to other scholars at a later date after the completion of this longitudinal study.
