# Supplementary material for: Longitudinal relations between interpartner aggression and internalizing symptoms among couples: The moderating role of sleep
Source: J Sleep Res. Author manuscript; Available in PMC 2025 Jan 25. (PMC11760190; doi:10.1111/jsr.14013)
Supplement: Supporting Material [file NIHMS2046795-supplement-Supporting_Material.pdf]

Supporting Information

Given that participants were in relationships, we considered actor-partner interdependence models (APIM) to assess research questions because of their ability to account for the interdependence of data (Kenny, 2018). Although this approach entails in the examination of partner effects and the estimation of paths non-pertinent to research questions, APIMs were fit in exploratory analyses. The findings were nearly identical to those reported in the Results (both direct relations and moderation results). Thus, when interdependence was taken into consideration, findings did not change.

Two APIMs were fit. In the first model depicted below, women’s sleep at T1 was examined as a moderator of relations between interpartner aggression against women at T1 and internalizing symptoms at T2. In the second model shown below, men’s sleep at T1 was examined as a moderator of relations between interpartner aggression against men at T1 and internalizing symptoms at T2.

Consistent with results reported in the manuscript (where separate models were fit for women and men), three interactions between interpartner aggression and sleep from the APIMs were significant and are depicted below.

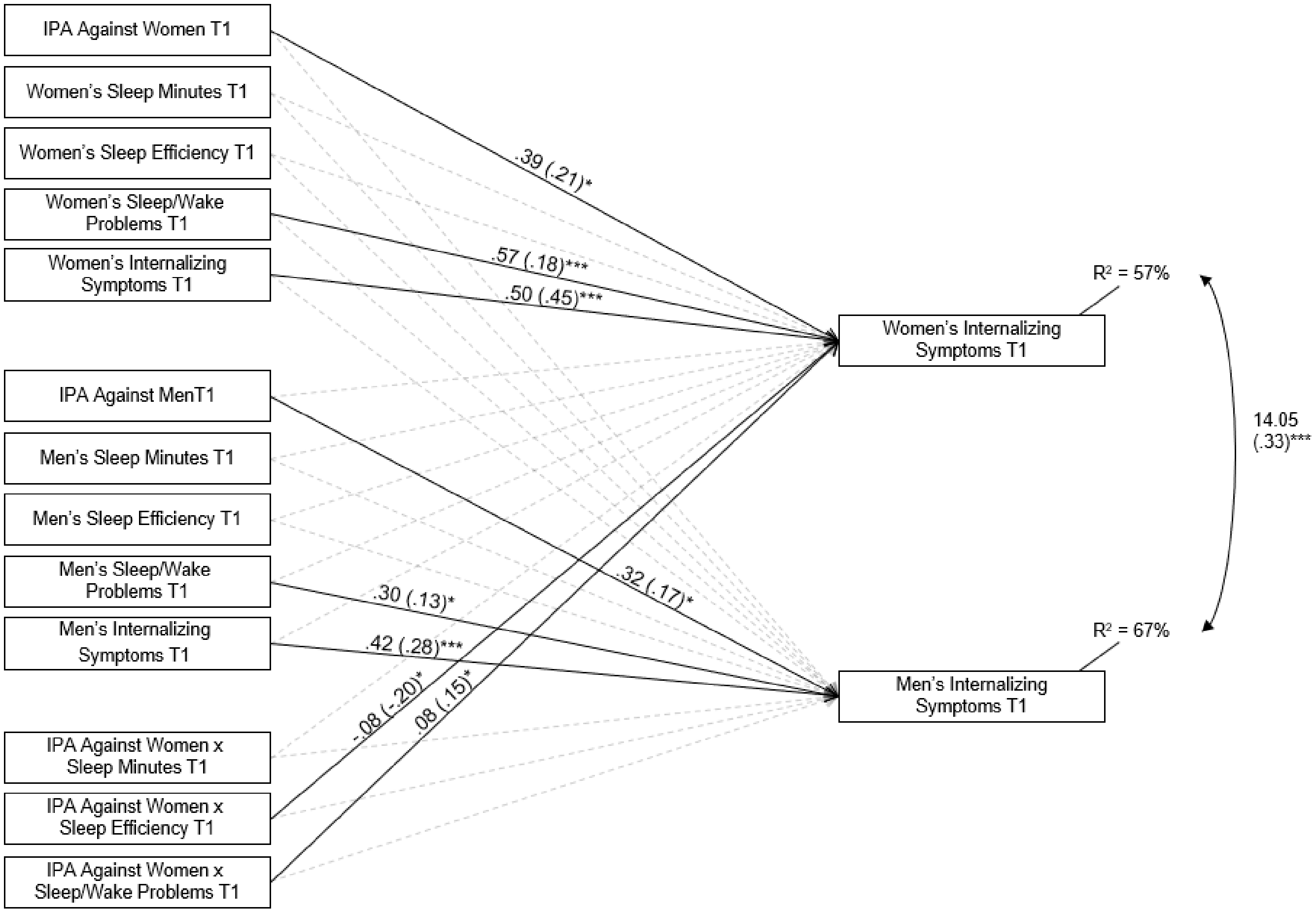

Actor-partner interdependence model fit to examine women's sleep at T1 as a moderator of relations between interpartner aggression (IPA) against women at T1 and internalizing symptoms at T2. For both women and men, race, age, and body mass index were included as control variables (the covariates and their associated paths are not depicted for clarity). Family socioeconomic status at T1 was also controlled. Statistically significant lines are solid and non-significant lines are dotted. Model fit: $\chi^2(120) = 116.21$*ns*; CFI = 1.00; RMSEA = .00*ns*, 95% CI [.00 to .03]. Exogenous variables that were significantly related were allowed to covary. Unstandardized and standardized coefficients (in parentheses) are included.

**p* < .05. ***p* < .01. ****p* < .001.

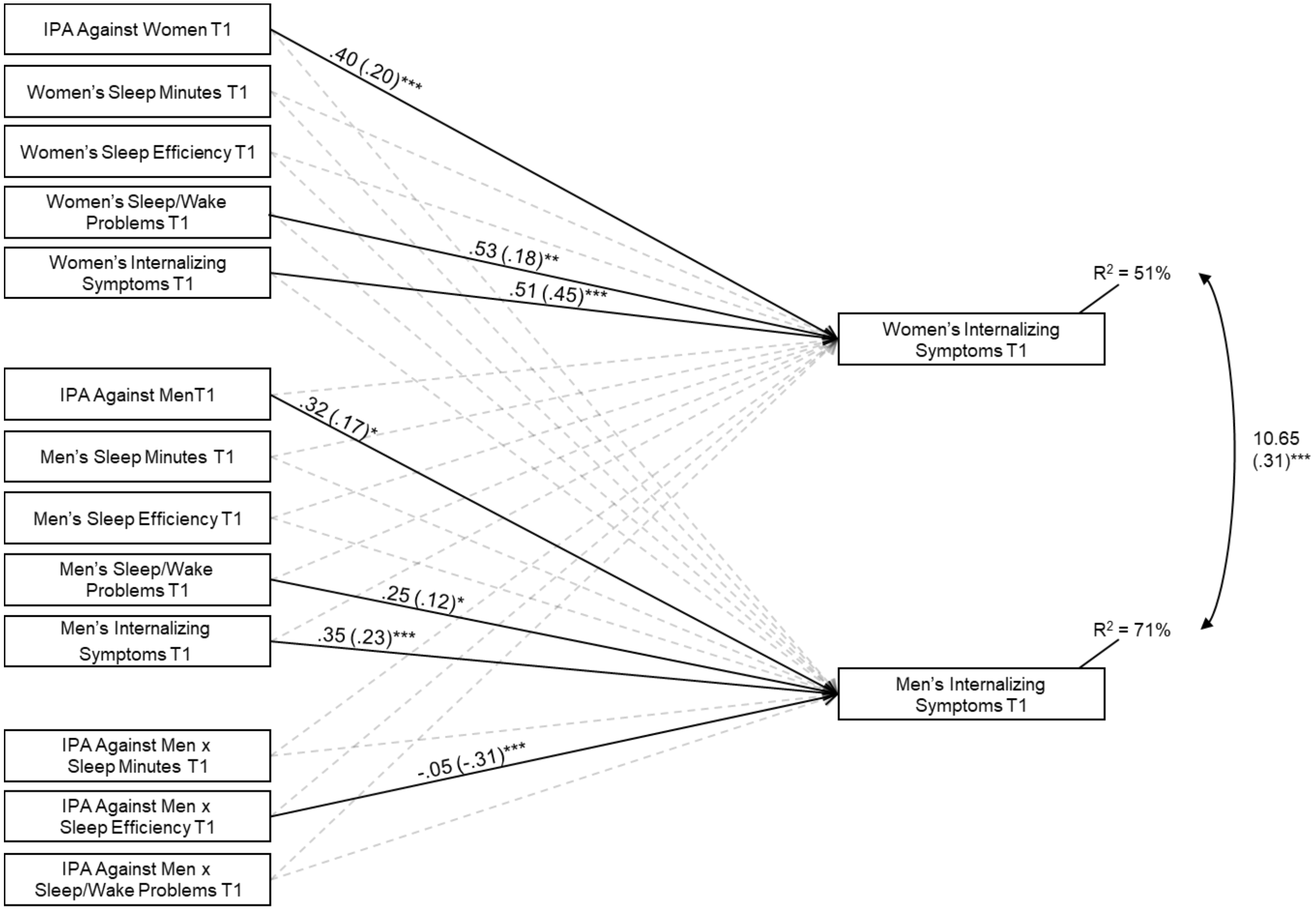

Actor-partner interdependence model fit to examine men's sleep at T1 as a moderator of relations between interpartner aggression (IPA) against men at T1 and internalizing symptoms at T2. For both women and men, race, age, and body mass index were included as control variables (the covariates and their associated paths are not depicted for clarity). Family socioeconomic status at T1 was also controlled. Model fit: $\chi^2(114) = 176.94$, $p < .001$; CFI = .95; RMSEA = .05*ns*, 95% CI [.03 to .07]. Exogenous variables that were significantly related were allowed to covary. Unstandardized and standardized coefficients (in parentheses) are included.

*$p < .05$. **$p < .01$. ***$p < .001$.

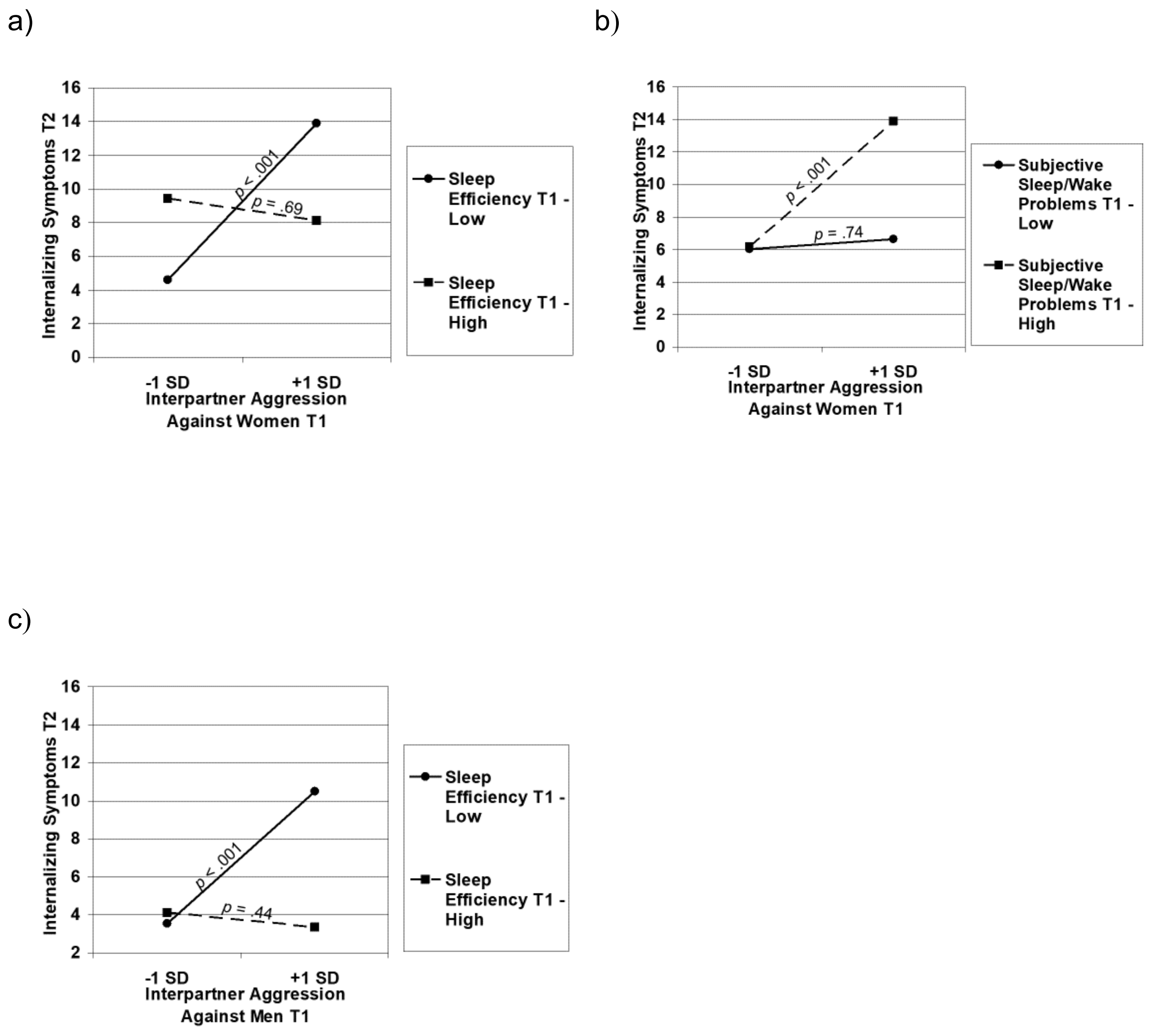

Depiction of the three significant interactions. a) Sleep efficiency at T1 as a moderator of relations between interpartner aggression against women at T1 and their internalizing symptoms at T2; b) Subjective sleep/wake problems at T1 as a moderator of relations between interpartner aggression against women at T1 and their internalizing symptoms at T2; c) Sleep efficiency at T1 as a moderator of relations between interpartner aggression against men at T1 their men's internalizing symptoms at T2.
